# PRIMARY SQUAMOUS CELL CARCINOMA OF THE COMMON BILE DUCT WITH LIVER METASTASES

**DOI:** 10.1590/0102-672020190004e1564

**Published:** 2021-05-14

**Authors:** Dhouha BACHA, Mohamed HAJRI, Wael FERJAOUI, Ghofrane TALBI, Lasaad GHARBI, Mohamed Taher KHALFALLAH, Sana ben SLAMA, Ahlem LAHMAR

**Affiliations:** 1 Department of General Surgery, Mongi Slim University Hospital,Faculty of Medicine of Tunis; 2 Department of Pathology, Mongi Slim University Hospital, Faculty of Medicine of Tunis

**Keywords:** Squamous cell carcinoma, Common bile duct, Liver metastasis, Carcinoma epidermoide, Ducto biliar comum, Metástase hepática

## INTRODUCTION

Cholangiocarcinoma is a cancer arising from thebiliary tree at any portion of the bile duct. Most of the tumors are diagnosed as adenocarcinomas^1^, whereas squamous cell carcinoma (SCC) are quite rare. Since the first case which was reported by Cabot and Painter^2^,only 35 cases of bile duct SCC have beenreported in the literature^3^. Being associated withe liver metastasis, SCC of bile duct is even more rare. To our knowledge, this is the sixth case documented in the literature^3^.

Here, we report the case of primary squamous cell carcinoma of the common bile duct (CBD) with metastatic liver.

## CASE REPORT

**FIGURE 1:**
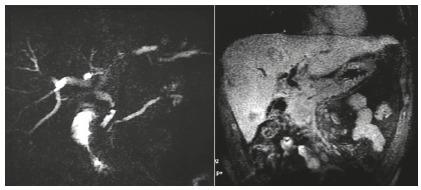
Magnetic resonance cholangiopancreatography showed stenosis of the distal CBD with liver metastasis

A 35-year-old man with no past medical history presented with epigastric pain, jaundice and general fatigue for one month. He had no history of cholelithiasis. Physical exam was normal except icteric skin and sclera. Laboratory data showed elevation of serum total bilirubin (376 µmol/l), direct fraction (318 µmol/l),*γ*-glutamyl transferase (900 u/l), alkaline phosphatase (300 u/l) and liver enzymes (ALT: 250 u/l, AST: 140 u/l). Tumours markers (cancer antigen 19-9 and carcinoembryonic antigen) were within normal limits. Abdominal ultrasonography showed gallbladder sludge, no stones in the CBD with increased diameter to 13 mm and distended intrahepatic bile ducts. Abdominal computed tomography scan revealed irregular wall*thickening*in the*distal*CBDwith dilatation of the intrahepatic and extrahepatic bile ducts. Otherwise, there weremultiple round lesions in many segments of liver suggestive of hepatic metastases.Magnetic resonance imaging showed a severe stenosis in the distal *portion of CBD*and revealed many liver lesions suggestive of liver metastases (Figure 1).

The diagnosis of a malignant extrahepatic tumour with liver metastasis has been retained.Percutaneous liver biopsy and echoendoscopic biopsy of the distal common bile duct were, therefore, decided upon.However, the patient presented hemodynamic instability related to severe acute cholangitis. He was, therefore, operated in emergency. Peroperative findings concluded to a tumor of the distal common bile duct with several liver metastases. Biliary drainage using T tube, bile duct brushing cytology and a liver biopsy were performed.

**FIGURE 2:**
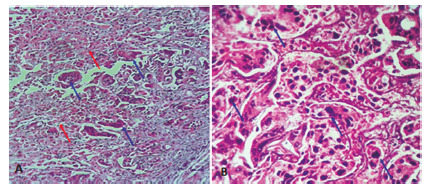
Pathological examination (H&E 40×), showing polyhedral cells (blue arrow) with irregular nuclei and with keratinization in the form of abundant eosinophilic cytoplasm: A) liver parenchyma; B) biliary brushing cytology

Histologically, the liver biopsy was composed of polyhedral to roundish cells *arranged in*irregular*heaps*and piles. The cells had irregular nuclei with keratinization in the form of abundant eosinophilic cytoplasm. Mitosis was found. Immunohistochemical evaluation was positive for cytokeratin 19 (Figure 2A). The same pathological cells were found in the biliary brushing cytology (Figure 2B).The patient was referred for palliative chemotherapy. He died of hepatic failure before starting chemotherapy one month after hospitalization.

## DISCUSSION

Our case is so interesting because of its rarity. It is the sixth case documented of SCC of the extrahepatic CBD associated with liver metastasis^3^. Indeed, biliary cancers represent 3% of digestive cancers^4^. It occurs, usually, in older patients^4^. Cholangiocarcinomas are the most frequent histological type (more than 90%)^5^. They are divided into intrahepatic and extra hepatic cholangiocarcinomas. In contrast, SCC is extremely rare. In the literature, we found 35 cases located in the extrahepatic bile duct^6^. Only six cases were associated with hepatic metastasis^3^. The pathogenesis of this rare entity is not elucidated to date.Many theories have been proposed. It may be due to continuous irritation (inflammatory stimulus for example) which leads tosquamous metaplasia of biliary epithelium and therefore to biliary SCC.hepatolithiasis, recurrent pyogenic cholangitis may be predisposing factors^6^and none of them were found in our patient. The symptomatology is similar to that of adenocarcinomas. Patients usually present with jaundice, abdominalpain, general fatigue and sometimes weight loss. Laboratory tests shows elevation of serum direct bilirubin and cholestasis as found in our case. Tumor markers such CA 19-9 and carcinoembryonicantigencanbeelevated. The radiologicalfeatures are usuallysimilar to those of adenocarcinomas.It appears as an irregular wall thickening with intense enhancement in the arterial phase. Distant metastases are rarely found^6^. In our case, several hepatic metastases were found on the computed tomography.Diagnosis can be made preoperatively using biopsy and histological examination. However, our patient was operated for severe acute cholangitis and the biopsy was made intraoperatively.Treatment strategies of SCC of CBD are not well established because of its rarity. Surgery is the main treatment withor without chemoradiotherapy (gemcitabine plus oxaliplatin or gemcitabine plus cisplatin)^5^. In case of hepatic metastases, palliative chemotherapy with gemcitabine is indicated.A review of the previously cases showed that the prognosis of this entity is extremely grave. Indeed, the median survival is estimated between 11 and 13 months^6^. The mortality rate was up to 63.3 %^5^.

## References

[1] Cleary SP, Dawson LA, Knox JJ, Gallinger S (2007). Cancer of the Gallbladder and Extrahepatic Bile Ducts. Curr Probl Surg.

[2] Cabot RC, Painter FM (1930). Case records of the Massachusetts General Hospital Case 16261: four months' jaundice and rectal pain. N Eng J Med.

[3] Goto T, Sasajima J, Koizumi K, Sugiyama Y, Kawamoto T, Fujibayashi S (2016). Primary Poorly Differentiated Squamous Cell Carcinoma of the Extrahepatic Bile Duct. Intern Med.

[4] Kang M, Kim NR, Chung DH, Cho HY, Park YH (2019). Squamous Cell Carcinoma of the Extrahepatic Common Hepatic Duct. J Pathol Transl Med. 2018/10/01.

[5] Khan SA, Thomas HC, Davidson BR, Taylor-Robinson SD (2005). Cholangiocarcinoma. The Lancet.

[6] Mori H, Kaneoka Y, Maeda A, Takayama Y, Fukami Y, Onoe S (2017). A perihilar bile duct squamous cell carcinoma treated by left hepaticlobe an caudate lobe resection, subtotal stomach preserving pancreatoduodenectomy,and portal vein resection. Jpn GastroenterolSurg.

